# Video microscopy as a tool to visualize cGMP effects on contractility and sperm transport in seminiferous tubules and the epididymal duct

**DOI:** 10.1186/2050-6511-14-S1-P44

**Published:** 2013-08-29

**Authors:** Andrea Mietens, Gerrit Eichner, Sabine Tasch, Caroline Feuerstacke, Ingrid Schneider-Hüther, Dieter Müller, Ralf Middendorff

**Affiliations:** 1 Institute of Anatomy and Cell Biology, Justus-Liebig University Giessen, 35385 Giessen, Germany; 2 Mathematical Institute, Justus-Liebig University Giessen, 35385 Giessen, Germany

## Background

Peritubular contractile cells within the testis and epididymis surround seminiferous tubules and the epididymal duct. These cells are crucial for the transport of sperm which only acquire motility and fertilizing capacity during transit from the testis to the distal epididymal duct. cGMP contributes to regulate smooth muscle cell function [1,2]. The role of cGMP, cGMP-hydrolysing PDE5 and potential effects of the PDE5 inhibitor sildenafil in testicular and epididymal smooth muscle cells, however, remains largely unknown despite the expanding use of sildenafil to treat erectile dysfunction and pulmonary hypertension.

## Results

Isolated epididymal duct segments and seminiferous tubules were studied by organ bath and time-lapse video microscopy to assess sperm transport and contractile activity. In all parts of the epididymis, regular contractions which elicited transport of sperm could be observed. Contraction frequency was reduced after the addition of cGMP-elevating agents sildenafil, nitric oxide (NO) or atrial natriuretic peptide (ANP) [3]. In seminiferous tubules, slow, irregular and very fine contractions of the tubular wall were observed without clear peristalsis. Wall movements were tracked over time by recording their varying grey values in defined regions of interest. After normalisation and detrending of these data, Fourier analysis could define a frequency spectrum characterizing this irregular contractile activity. Elevating cGMP resulted in a shift of the frequency spectrum towards lower frequencies.

Western blotting, immunohistochemistry and RT-PCR using laser-dissected contractile cells confirmed expression of the corresponding cGMP pathway components. PDE5 activity was assessed using a cGMP-ELISA and sildenafil dose-dependently increased cGMP.

In a rat model of chronic sildenafil treatment, PDE5 expression, contractility and sperm transport remained unaltered.

## Conclusion

Video microscopy allows to visualize contractile activity and sperm transport in seminiferous tubules and the epididymal duct. Distinct patterns of spontaneous contractile activity are observed in both organs. In the epididymis, regular spontaneous and peristaltic contractions elicit movement of sperm and contractile frequency is reduced by increasing cGMP. In contrast, seminiferous tubules, show an irregular contractile activity of smaller amplitude without clear peristalsis. Our results suggest that this irregular activity can be characterized by a spectrum of frequencies generated by Fourier analysis and that increasing cGMP slows down spontaneous contractile activity as indicated by a shift towards lower frequencies.
